# Supplementary material for: Invasion preferences suggest a possible role for Plasmodium falciparum parasites in the expansion of Duffy negativity in West and Central Africa
Source: Mol Biol Evol. 2026 Feb 3;43(2):msag033. doi: 10.1093/molbev/msag033 (PMC12930091; doi:10.1093/molbev/msag033)
Supplement: msag033_Supplementary_Data [file msag033_supplementary_data.pdf]

Supplementary Information

Invasion preferences suggest a possible role for *Plasmodium falciparum* parasites in the expansion of Duffy negativity in West and Central Africa

Balanding Manneh[1], Viola Introini[1,2,4], James Reed[3], Madalina Rotariu[3], Robin Antrobus[1], Pietro Cicuta[2], Michael P. Weekes[1], Bridget S. Penman[5], Julian C. Rayner[1]

[1]Cambridge Institute for Medical Research, University of Cambridge, Cambridge, UK

[2]Cavendish Laboratory, University of Cambridge, Cambridge, UK

[3]Research Donors, London, UK

[4]European Molecular Biology Laboratory, Barcelona, Spain

[5]Department of Biology, University of Oxford, Oxford, UK

Supplementary Figures

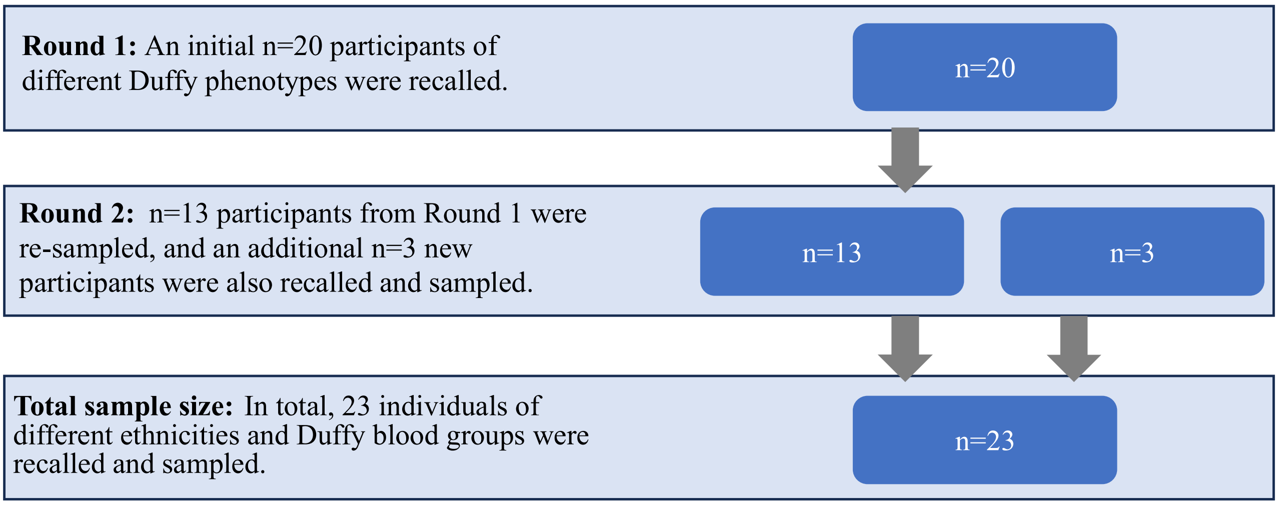

Figure S1: Sample collection and study design. The Duffy blood samples were collected in two recall-by-genotype study rounds in 2021 (round 1) and 2022 (round 2), from 20 participants in round 1 and 16 participants in round 2 (13 of the 20 participants from round 1 were re-sampled, and three new participants were added). The round 1 samples were received in batches of five (i.e., five bi-weekly batches and five samples per batch), and only three of the four major Duffy blood groups were represented in each batch, while in round 2, samples were collected in four bi-weekly batches (four samples per batch) and all four Duffy phenotypes were represented in each of the four batches. The presence of all four Duffy blood groups in a batch was useful for the preference assays as it meant that RBCs from all four samples could be labelled with different fluorescent dyes and incubated together with malaria parasites in the same well, allowing for the comparison of invasion into all four different RBC samples simultaneously.

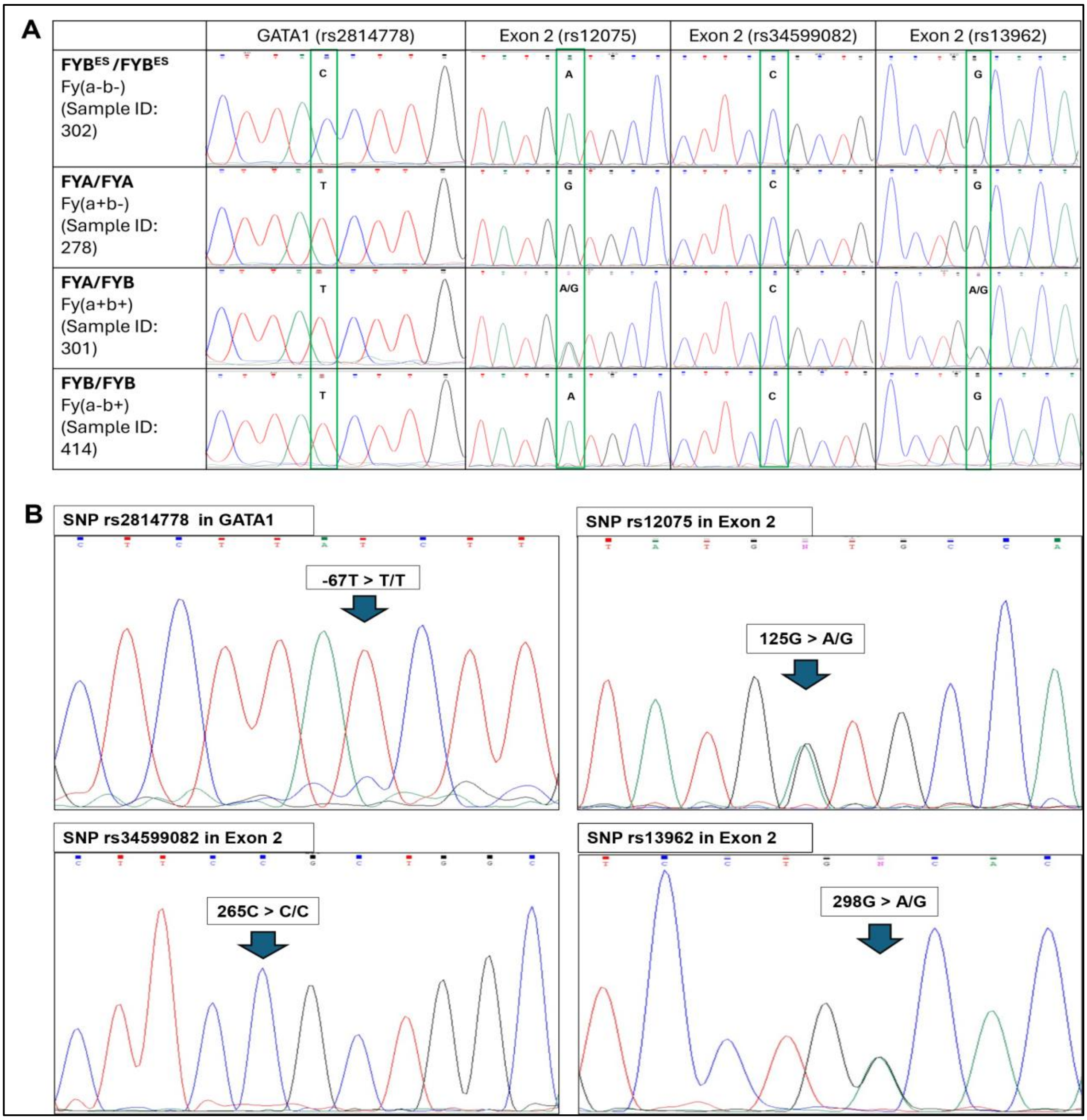

Figure S2: Segments of chromatograms of Duffy-positive and -negative samples.
(**A**) Chromatogram of four Duffy samples showing the GATA1 mutation (-67 T>C; rs2814778) for a Duffy-negative sample (sample 302), and the wildtype SNP (-67 T>T; rs2814778) for homozygous FYA sample (FYA/FYA; sample 278), heterozygous (FYA/FYB; sample 301), and homozygous FYB sample (FYB/FYB; sample 414). The positions of all SNPs, both in the GATA1 (rs2814778) and Exon 2 (rs12075; rs34599082; and rs13962), are highlighted in green rectangles for all samples.
(**B**) Segments of a chromatogram from a Duffy-positive sample (sample 559) in round 2 that was genotyped as FYA/FYB (heterozygous), showing SNP locations in the GATA1 and Exon 2 regions of the gene. The agglutination test showed that the donor has the Fy(a+b-) phenotype; however, the sequencing results above showed that the individual has no mutation in GATA1 (-67 T> T/T; wildtype). The SNP location (125G>A/G; rs12075) determines the presence of the FYA or FYB alleles and their corresponding phenotypes. Two peaks of similar intensity are seen at position 125 in Exon 2, meaning the individual has the co-dominant FYA and the FYB alleles at this locus (i.e., 125G > A/G) and would express both Fy^a/Fy^b antigens and the phenotype Fy(a+b+). No mutation is seen at position 265, the third SNP location (rs34599082). A mutation is present at position 298 in Exon 2, and two peaks of relatively similar intensities are seen, indicating that the individual has both the FYA and FYB alleles at this locus, and the donor was assigned the Fy(a+b+) phenotype.

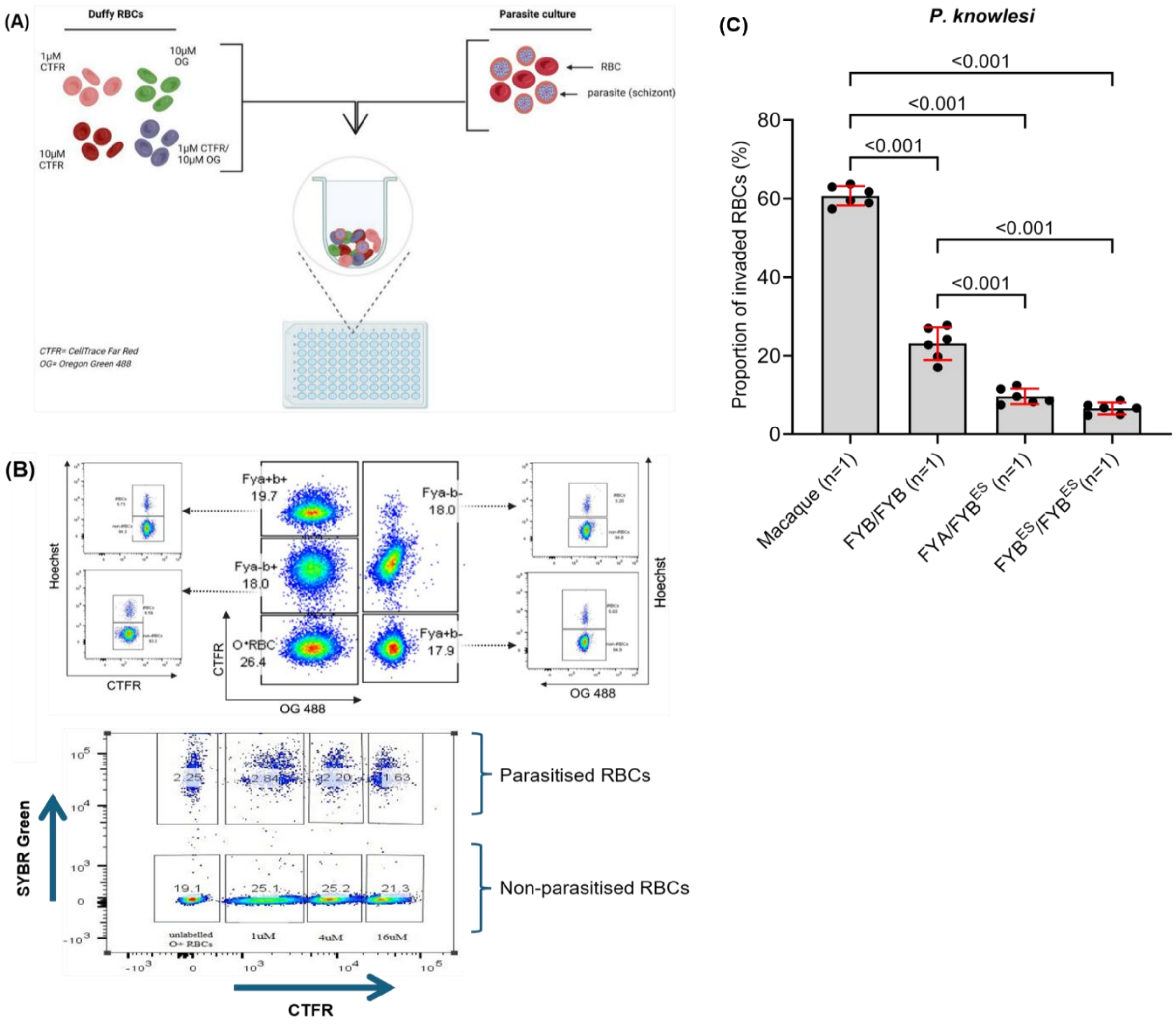

Figure S3: The *in vitro* parasite preference assay.

(**A**) Schematic of the *in vitro* preference assay based on an earlier version (Theron et al. 2018).

(**B**) Top panel - gating strategy used for the round 2 samples (RBCs from all 4 Duffy phenotypes were incubated together with parasites in a single well), showing the proportion of invaded RBCs by *P. falciparum* in a single well of a preference assay after debris and doublets were excluded. Each of the Duffy RBCs contained both parasitised and non-parasitised RBCs and was gated using the fluorescent intensities of CTFR vs Hoechst (to quantify CTFR-labelled parasitised RBCs) and OG vs Hoechst (to quantify OG-labelled parasitised RBCs). Bottom panel -gating strategy used for round 1 samples, showing the parasitised and non-parasitised RBCs after debris and doublets were excluded. The RBCs were labelled with increasing concentrations of CTFR, and the percentage of parasitised RBCs in each labelled RBC group was determined using the fluorescence intensity of CTFR vs SYBR Green dyes.

(**C**) We assessed the sensitivity of the above preference assay using Macaque and human RBCs prior to analysing the Duffy samples. The macaque RBCs (of unknown genotype) were preferentially invaded over the human RBCs, and among the human RBCs, the FYB/FYB RBCs were preferentially invaded over the other genotypes. On average, 60% (±2.5) of the Macaque RBCs were invaded compared to 23.1% (±4.2) for FYB/FYB, 9.7% (±2.0) for FYA/FYB^{ES}, and 6.6% (±1.5) for FYB^{ES}/FYB^{ES} RBCs. These data clearly show that the preference assay is highly sensitive and able to reliably detect even minor differences in the invasion preference of human malaria parasites. The assay was performed as described in the Methods, using the same *P. knowlesi* parasites and human RBCs, and each assay was performed in triplicate, resulting in six replicates for each donor sample. Statistical comparisons between the samples were performed using a one-way ANOVA, and a Tukey's test was used to correct for multiple comparisons. The mean, standard deviation, and statistically significant differences are shown for each sample.

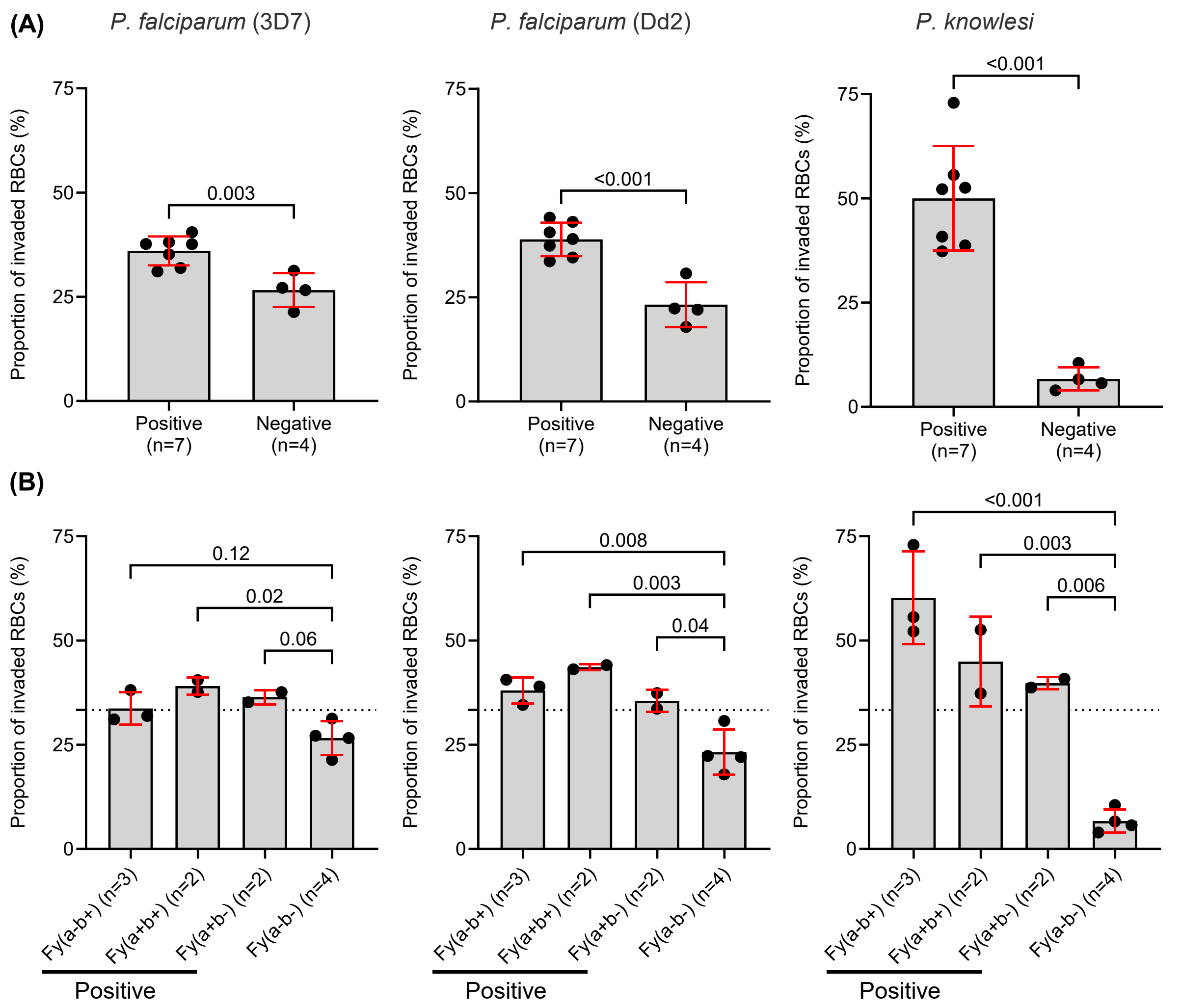

Figure S4: The invasion preference of malaria parasites for Duffy RBC groups in round 1. In round 1, only three Duffy phenotypes per batch were compared at a time in the preference assay, as shown in Figure S3B, and all parasites showed a clear preference for RBCs from Duffy-positive individuals.

(**A**) The preference of *P. falciparum* parasites was skewed towards Duffy-positive RBCs and away from Duffy-negative RBCs. When grouped by Duffy status (i.e., Duffy-positive vs Duffy-negative), the average proportion of invaded RBCs by 3D7 parasites was 36.0% (±3.5) for the Duffy-positive and 26.6% (±4.1) for the Duffy-negative RBCs, respectively. The average invasion for Dd2 parasites was 38.9% (±4.0) for Duffy-positive and 23.2% (±5.4) for Duffy-negative RBCs. As expected, invasion of the Duffy-negative RBCs by *P. knowlesi* was significantly reduced relative to Duffy-positive RBCs; the average proportion of invaded RBCs was 50.0% (±12.5) for Duffy-positive compared to 6.7% (±2.8) for Duffy-negative RBCs.

(**B**) As several Duffy genotypes/phenotypes are represented in the Duffy-positive samples, we re-analysed the data to compare invasion rates in all four Duffy phenotypes. The average proportion of invaded RBCs with 3D7 was 33.7% (±3.9), 39.1% (±2.0), 36.4% (±1.7), and 26.6% (±4.1) for Fy(a-b+), Fy(a+b+), Fy(a+b-), and Fy(a-b-) RBCs, respectively. The Dd2 parasites also showed an invasion preference for Duffy-positive RBCs over Duffy-negative RBCs with an average invasion of 38.0% (±3.1), 43.6% (±0.7), 35.5% (±2.7) for Fy(a-b+), Fy(a+b+), and Fy(a+b-) RBCs, respectively, compared to 23.2% (±5.4) for the Duffy-negative RBCs. The average proportion of invaded RBCs for the *P. knowlesi* parasites was 60.2% (±11.1), 44.9% (±10.8), and 39.8% (±1.5) for the Fy(a-b+), Fy(a+b+), and Fy(a+b-) RBCs, respectively, compared to 6.7% (±2.8) for the Duffy-negative RBCs. Statistical comparison for data in (A) was carried out using a two-tailed t-test, and comparisons between the four RBC groups in (B) were carried out using One-way ANOVA, followed by pairwise comparisons using Tukey's HSD (honestly significant difference) test. The mean and standard deviation are shown for each group.

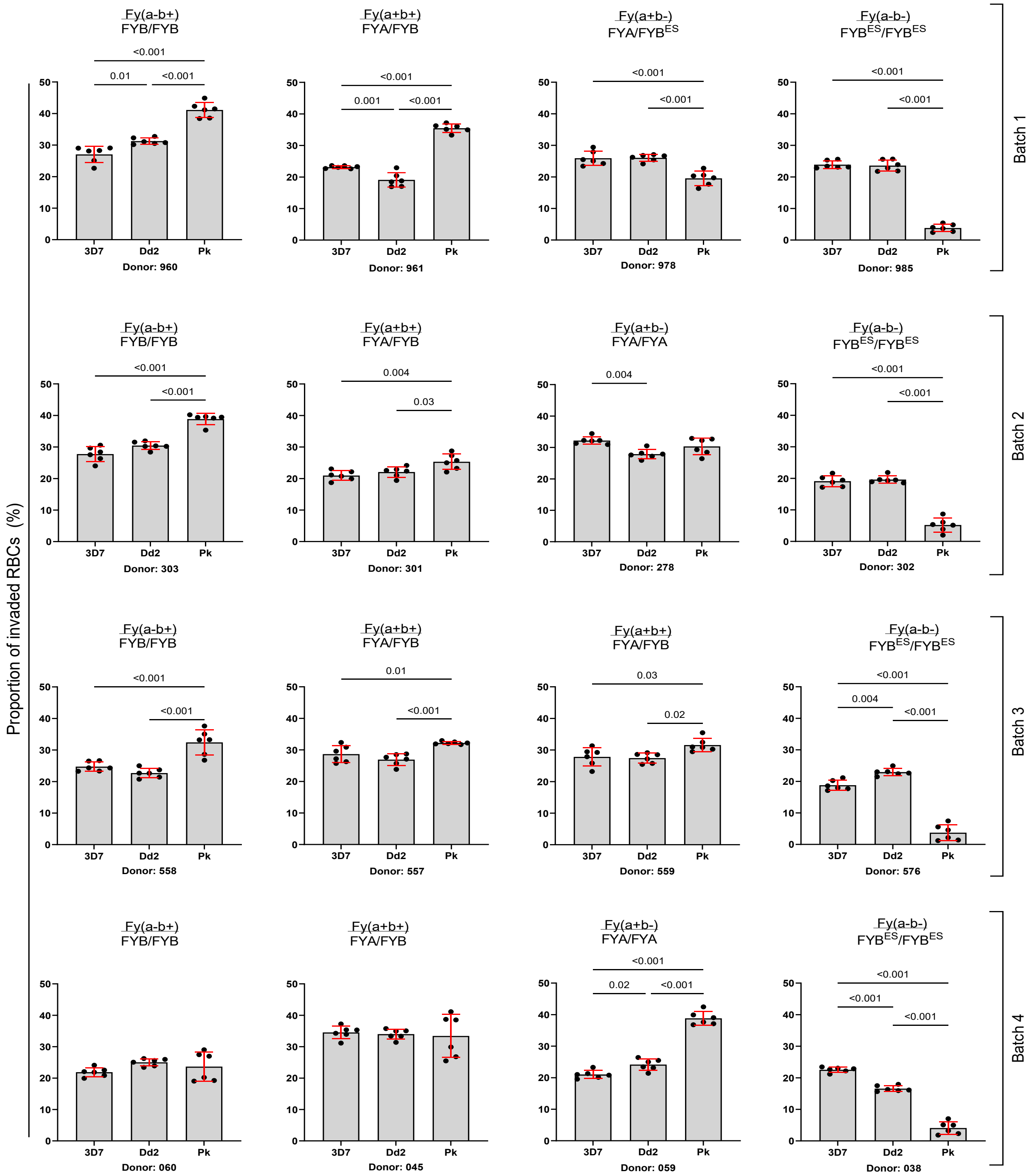

Figure S5: The invasion rates of three malaria parasites in donor RBC sample tested in round 2. Each donor RBC sample was tested with *P. falciparum* (3D7 and Dd2 strains), and *P. knowlesi* (Pk) parasites in triplicate, and each assay was duplicated to produce six replicates per donor; all six replicates are shown. The corresponding Duffy phenotype and genotype are listed above each sample. In Batch 3, the agglutination test showed that donor 559 has the Fy(a+b-) phenotype; however, the genotyping showed that the donor is FYA/FYB (heterozygous) (Figure S4B). The bar plots are coloured based on Duffy RBC phenotype (blood group), and the *y-axis* represents the proportion of invaded RBCs in each sample by the tested parasites. Statistical comparisons between the groups were carried out using One-way ANOVA, followed by pairwise comparisons using Tukey's test. The mean and standard deviations are shown for each parasite strain/species, and all statistically significant *P* values (i.e. $P \leq 0.05$) are shown.

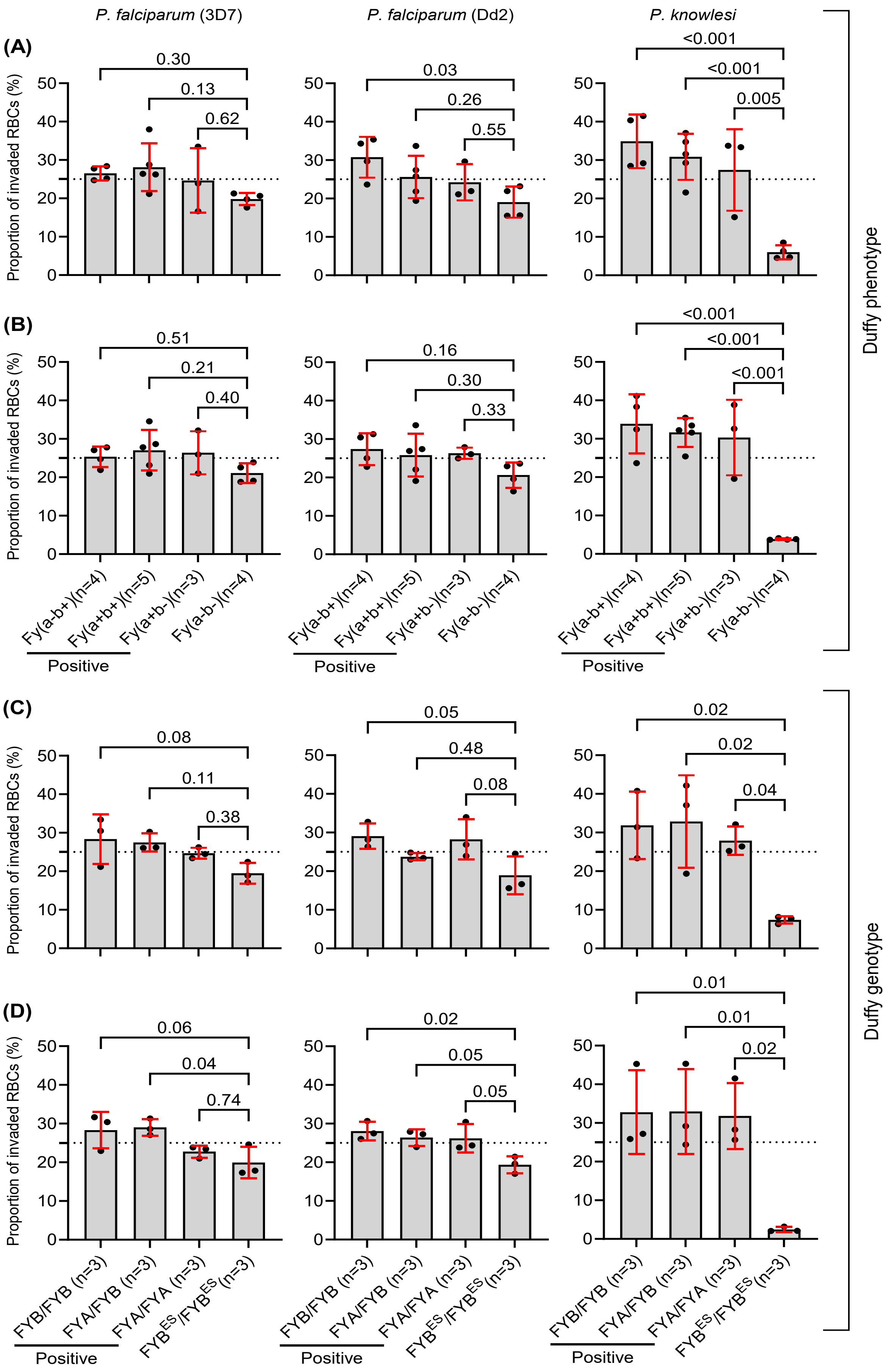

P. falciparum (3D7)
P. falciparum (Dd2)
P. knowlesi
(A)
(B)
(C)
(D)
Proportion of invaded RBCs (%)
0.30
0.13
0.62
0.03
0.26
0.55
<0.001
<0.001
0.005
0.51
0.21
0.40
0.16
0.30
0.33
<0.001
<0.001
<0.001
Fy(a-b+)(n=4)
Fy(a+b+)(n=5)
Fy(a+b-)(n=3)
Fy(a-b-)(n=4)
Positive
Duffy phenotype
0.08
0.11
0.38
0.05
0.48
0.08
0.02
0.02
0.04
0.06
0.04
0.74
0.02
0.05
0.05
0.01
0.01
0.02
FYB/FYB (n=3)
FYA/FYB (n=3)
FYA/FYA (n=3)
FYB^ES/FYB^ES (n=3)
Positive
Duffy genotype

Figure S6: Effect of Duffy phenotype and genotype on RBC invasion by *P. falciparum* and *P. knowlesi* parasites.

(**A**) The Duffy phenotypes of the grouped data presented in Figure 1A and C.

(**B**) The Duffy phenotypes of the grouped data presented in Figure 1B and D.

(**C**) To assess the impact of Duffy genotype on the invasion preference of the malaria parasites, we performed additional sets of preference assays using the same RBC samples and parasites as in Figure 1 and Figure S4, to compare invasion only by genotype rather than phenotype.

(**D**) The Duffy genotypes were also tested in invasion assays as in (C) above. All six replicates are averaged for each donor. Statistical comparisons were performed using a one-way ANOVA, followed by Tukey's test.

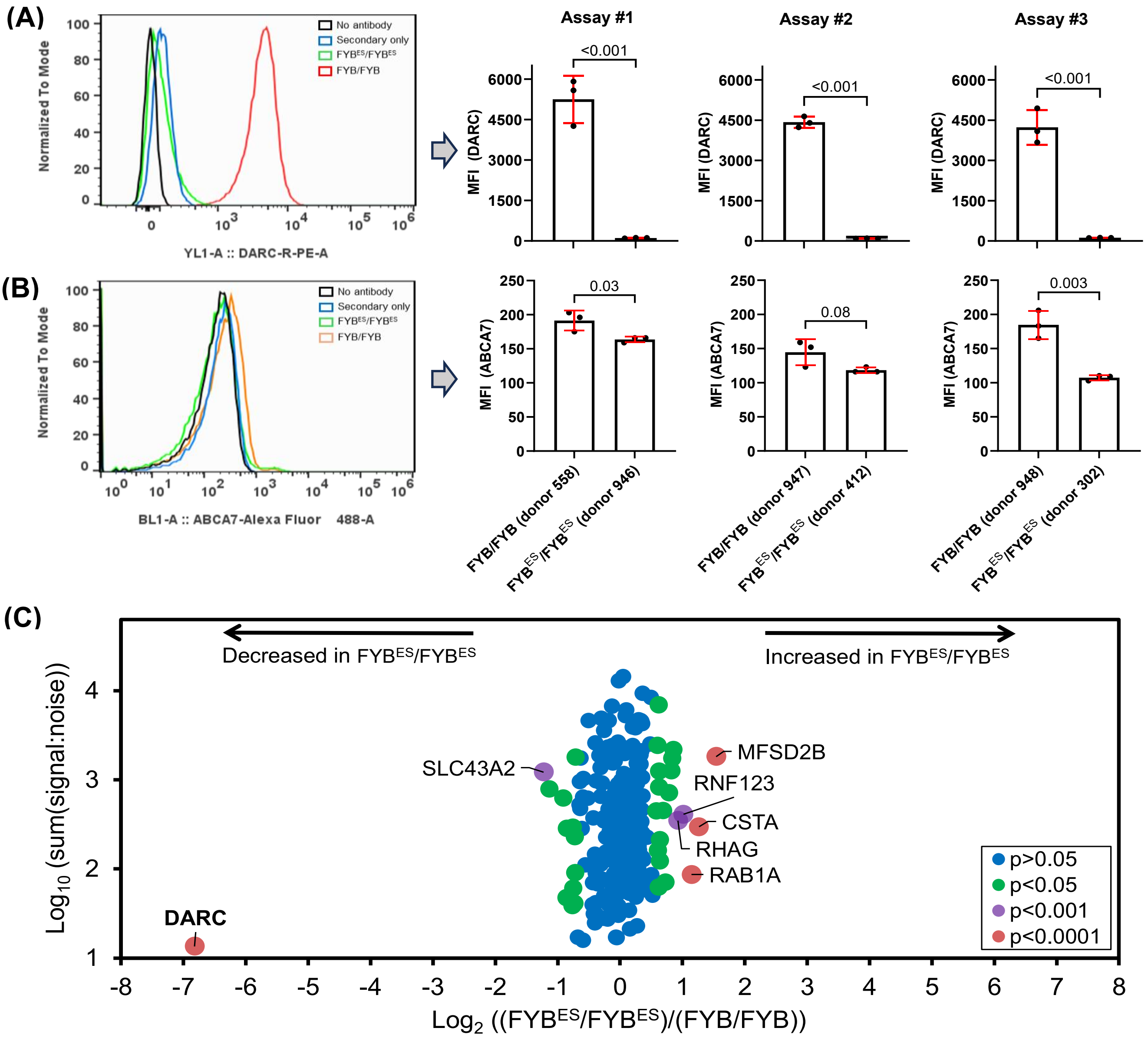

Figure S7: The relative abundance of DARC, ABCA7, and other surface proteins on FYB/FYB and FYB^{ES}/FYB^{ES} RBCs as quantified by flow cytometry and mass spectrometry.

(A) DARC antigen (Fy^6) expression was quantified on FYB/FYB (n=3) and FYB^{ES}/FYB^{ES} (n=3) RBCs by flow cytometry. As expected, significant differences were found between the two genotypes.

(B) ABCA7 expression was also determined by flow cytometry, and statistically significant differences were found between the two genotypes. The flow cytometry results for both DARC and ABCA7 are consistent with the mass spectrometry results in Figure 3. Each sample was tested in triplicate across three independent assays. Statistical comparisons were carried out using a two-tailed t-test; the mean and standard deviations are shown for each sample; MFI = median fluorescence intensity.

(C) The preference and invasion data showed that all tested malaria parasites had a preference for the FYB/FYB relative to FYB^{ES}/FYB^{ES} RBCs and the other tested genotypes (Figure 1C-D, Figure S6C-D). We therefore re-analysed the proteomics data in Figure 3A to compare the relative abundance of surface proteins on FYB/FYB (n=3) and FYB^{ES}/FYB^{ES} (n=4) RBCs. We found that DARC was the single most downregulated protein in FYB^{ES}/FYB^{ES} RBCs, but the ABCA7 signal was absent. The *x-axis* shows the fold change, which was calculated for each protein using the signal-to-noise ratio of FYB^{ES}/FYB^{ES} to FYB/FYB RBCs, and the y-axis shows the summed signal-to-noise ratio of FYB^{ES}/FYB^{ES} and FYB/FYB RBCs. A two-tailed t-test was used to determine the level of significance and estimate *P* values, which were corrected for multiple hypothesis testing using the Benjamini–Hochberg method.

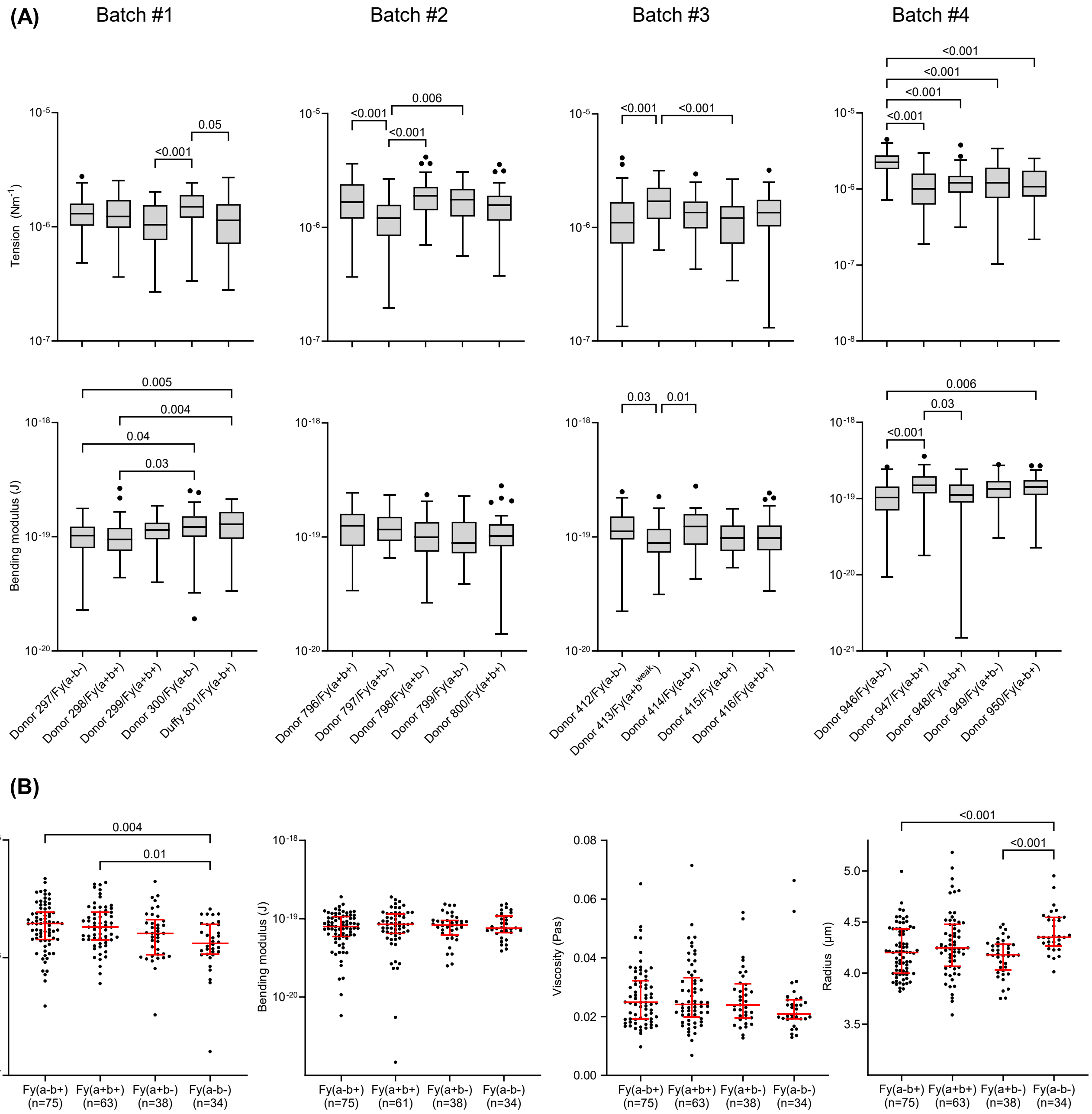

Figure S8: The biophysical properties of Duffy-positive and negative RBCs collected in both rounds.
(A) The data for tension and bending in Figure 2A (collected in round 1) were re-analysed for individual sample donors, to determine both donor/phenotype and batch variability.
(B) Flickering analysis of Duffy RBC samples collected in round 2. The Duffy RBC samples analysed in this study were collected in two rounds, as summarised in Figure S1**.** As all samples except three (3) samples were initially collected in round 1 and analysed, only a randomly selected subset of the samples (n=6) was therefore analysed in round 2 – (Fy(a-b+)(n=2), Fy(a+b+)(n=2), Fy(a+b-)(n=1), and Fy(a-b-)(n=1). Statistical significance for both Figure A and B was determined by a Kruskal-Wallis test, and differences were corrected for multiple comparisons using Dunn's test. The median, IQR, and statistically significant differences are shown for each phenotype.

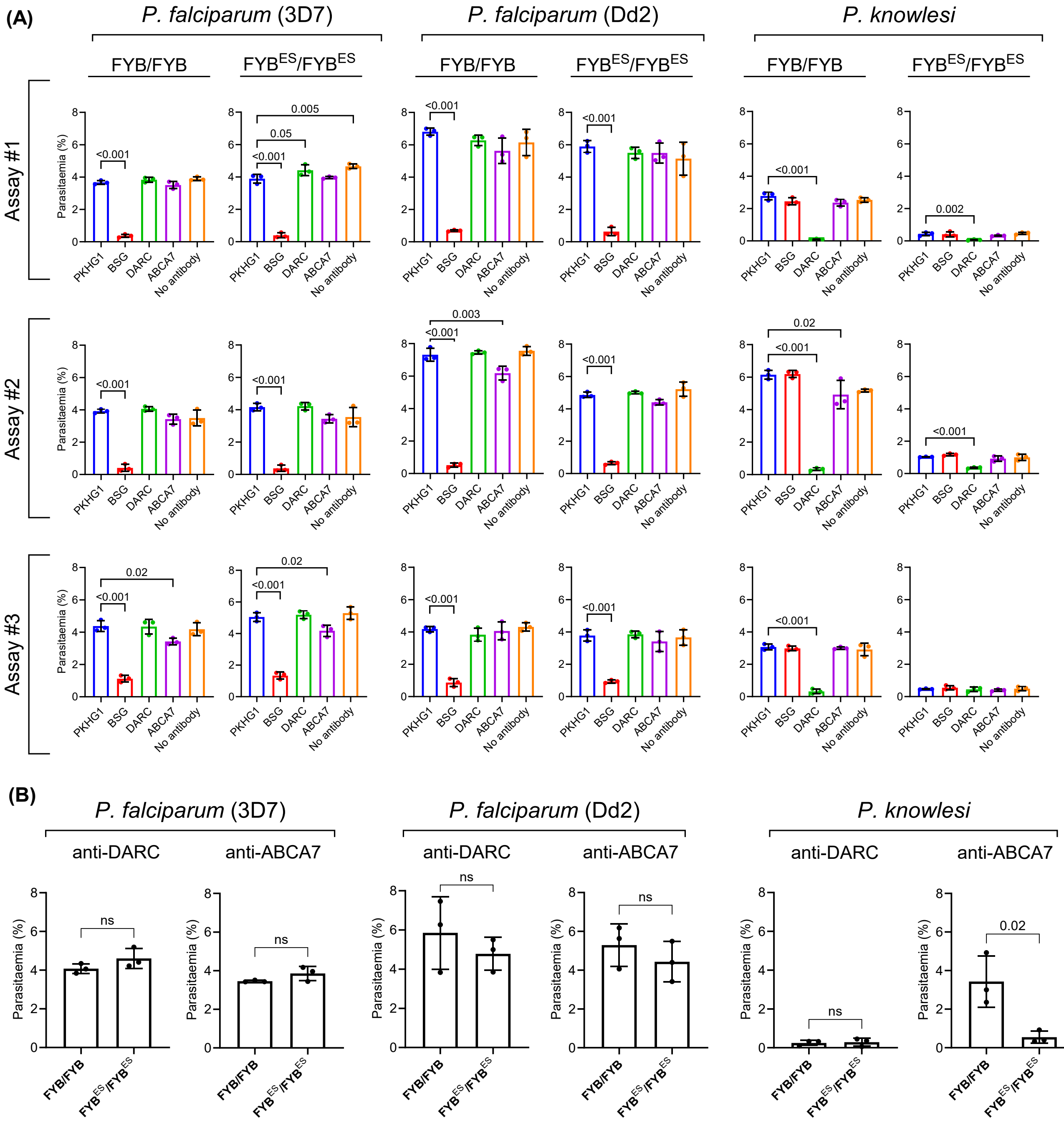

Figure S9: Effect of a panel of four antibodies on the invasion of Duffy-positive and -negative RBCs by *P. falciparum* and *P. knowlesi* parasites.

(A) The invasion of FYB/FYB (n=3) and FYB^{ES}/FYB^{ES} (n=3) RBCs in the presence and absence of a panel of four antibodies was quantified by flow cytometry. Each RBC was tested in triplicate against all antibodies using both *P. falciparum* (3D7 and Dd2) and *P. knowlesi* parasites; all replicates are shown for each treatment. Each assay was performed a week apart using the same parasites and a new pair of FYB/FYB and FYB^{ES}/FYB^{ES} samples. Statistical comparisons were performed using a one-way ANOVA, followed by pairwise comparisons using Dunnett's test.

(B) Data in Figure A above were re-analysed to compare invasion rates between Duffy-positive and -negative RBCs in the presence of anti-DARC and anti-ABCA7. Statistical comparison was performed using a two-tailed t-test. The average of replicates for each of the 3 tested FYB/FYB and FYB^{ES}/FYB^{ES} samples, standard deviation, and mean are shown.

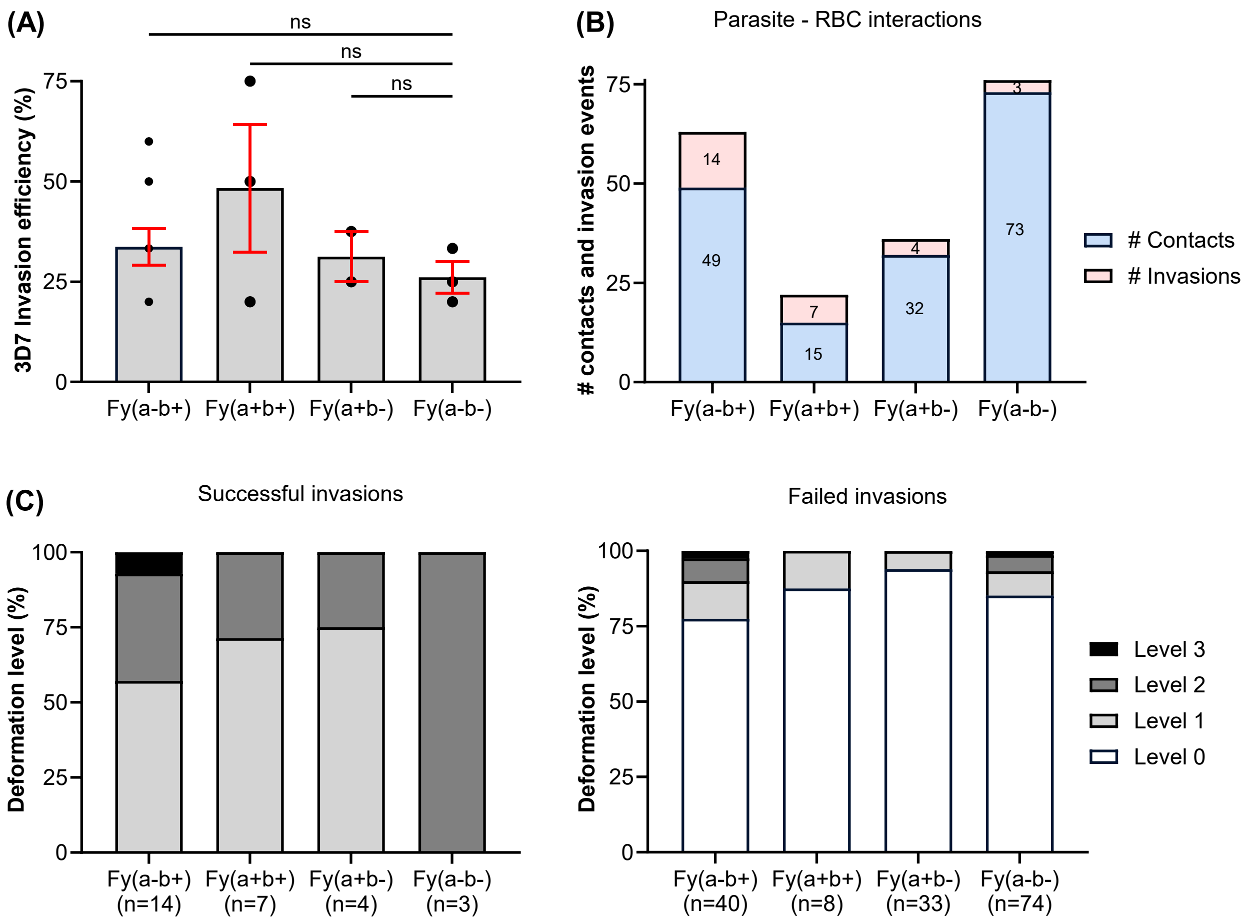

Figure S10: Invasion efficiency and deformation of Duffy-positive and -negative RBCs by 3D7 parasites. (**A**) The invasion efficiency of 3D7 parasites was quantified by video microscopy. The following RBC groups were analysed: Fy(a-b+)(n=3); Fy(a+b+)(n=2); Fy(a+b-)(n=2); and Fy(a-b-)(n=3). The results showed no statistically significant differences in invasion efficiency between the RBC samples. Statistical comparisons were carried out using One-way ANOVA, and Tukey's test was used to determine the significant differences between the groups. The bar plot shows the mean and standard error of the mean (SEM) for each Duffy RBC group, and *ns* = not statistically significant – i.e., $P > 0.05$.
(**B**) The number of times merozoites came into contact with RBCs and successfully invaded is shown for each Duffy blood group. The ratio of successful invasions and the number of merozoites that came in contact with RBCs was used to determine the invasion efficiency in (A) above.
(**C**) The deformation levels of successful and failed invasion events were also determined. The number below each phenotype 'n' represents the number of merozoites that deformed the RBCs and either failed or successfully invaded each Duffy phenotype.

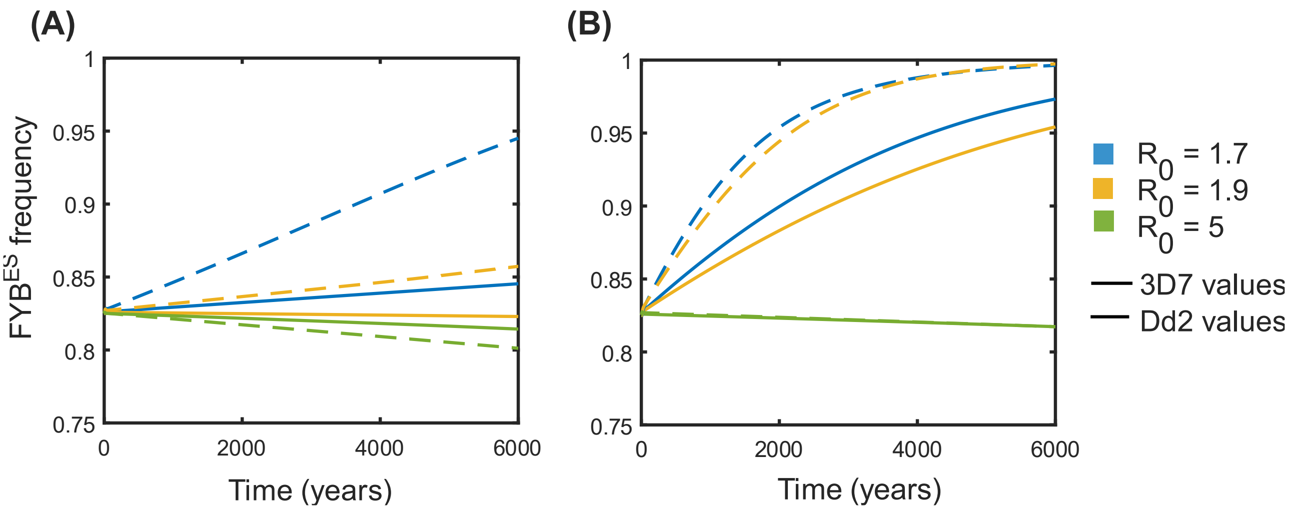

Figure S11: Sensitivity analysis of Figure 5. Figures (A) and (B) both use a starting frequency of 0.825 FYB^{ES}. In panel (A) θ = 1/5, α_1 =0.04 and α_2 = 0.00005. In (B), θ = 1/20, α_1 = 0.005 and α_2 = 0.00005. The relationship that we predict (i.e. that *P. falciparum* is most likely to elevate FYB^{ES} frequencies at relatively low levels of transmission) is not dependent on the exact parameter values that were used in Table S7. To illustrate this, Figure S11 re-generates two new versions of Figure 5A, but using different malaria mortality rates and assuming that immunity to *P. falciparum* virulence is gained even more rapidly than in Figure 5. The rates of allele frequency change are different, but the pattern that FYB^{ES} frequencies increase more rapidly with lower *P. falciparum* transmission remains.

Supplementary Tables

Table S1: Demographic characteristics and sample parameters of study participants.

**Round 1**					
**Characteristics**	**Fy(a-b+)**	**Fy(a+b+)**	**Fy(a+b-)**	**Fy(a-b-)**	**Fy(a+b^w)**
Sample size (n)	6	5	3	5	1
Age in years (sd)	44 (13.78)	27.4 (5.13)	30.7 (7.51)	37 (15.15)	24
Sex (M/F)	3/3	3/2	2/1	3/2	1/0
White blood cell count (10^9 L) (sd)	6.25 (2.51)	7.99 (2.70)	4.91 (0.66)	5.11 (0.96)	8.34
Red blood cell count (10^{12} L) (sd)	4.73 (0.39)	5.01 (0.36)	4.84 (0.34)	4.64 (0.47)	6.0
Haemoglobin (g/L) (sd)	142.7 (8.02)	137.8 (16.54)	147 (8.0)	129.6 (13.22)	117
Haematocrit (L/L) (sd)	0.429 (0.023)	0.426 (0.038)	0.438 (0.019)	0.391 (0.042)	0.359
Mean cell volume (fL) (sd)	91.15 (4.05)	85 (2.67)	90.2 (4.35)	84.48 (7.59)	59.8
Mean cell haemoglobin (pg) (sd)	30.23 (1.46)	27.44 (1.65)	30.27 (1.54)	28.06 (3.21)	19.5
Mean cell haemoglobin concentration (g/L) (sd)	332 (6.07)	322.6 (11.48)	335.7 (3.06)	332 (17.35)	326
Platelet (10^9 L) (sd)	267 (111.8)	265 (55.65)	194 (8.96)	195 (58.48)	196
RBC distribution width (%) (sd)	12.85 (0.75)	13.66 (1.42)	12 (0.44)	13.84 (2.56)	18.5
**Round 2**					
Sample size (n)	4	5	3	4	-
Age in years (sd)	46.5 (9.95)	27.2 (4.09)	31.33 (8.08)	30.5 (13.03)	-
Sex (M/F)	1/3	4/1	2/1	3/1	-
White blood cell count (10^9 L) (sd)	6.91 (2.38)	6.58 (2.32)	5.08 (0.77)	4.66 (0.89)	-
Red blood cell count (10^{12} L) (sd)	4.69 (0.29)	5.04 (0.37)	4.84 (0.42)	5.14 (0.46)	-
Haemoglobin (g/L) (sd)	139.00 (4.24)	141.60 (13.26)	144.67 (6.81)	136.50 (11.21)	-
Haematocrit (L/L) (sd)	0.42 (0.01)	0.43 (0.03)	0.44 (0.02)	0.43 (0.02)	-
Mean cell volume (fL) (sd)	90.55 (4.23)	86.12 (3.18)	91.17 (3.37)	83.88 (7.31)	-
Mean cell haemoglobin (pg) (sd)	29.70 (1.16)	28.14 (1.37)	30.00 (1.39)	26.68 (3.01)	-
Mean cell haemoglobin concentration (g/L) (sd)	328.25 (4.35)	326.40 (8.41)	328.67 (3.21)	317.75 (9.91)	-
Platelet (10^9 L) (sd)	267 (116.72)	235.20 (52.10)	196.67 (21.36)	205.00 (56.02)	-
RBC distribution width (%) (sd)	13.05 (0.42)	12.92 (1.27)	12.03 (0.55)	14.50 (3.04)	-

The table shows the demographic characteristics and blood sample parameters of study participants from both round 1 and round 2 at the time of sample collection. The mean values and standard deviations (sd) are shown for the age variable and RBC indices for each Duffy blood group. The sample size for each Duffy blood group is represented by the letter n in parenthesis (n); for the sex variable, F = female and M = male; and W = weak expression of the Fyb antigen on RBCs.

Table S2: Duffy genotypes and phenotypes of study participants in both rounds 1 and 2.

Sample ID	Ethnicity	ABO	ABCA7	Genotype	Phenotype	Fy Antigen	Year sampled
950/960	Asian (South)	A	Wildtype	FYB/FYB	Fy(a-b+)	Fyb+	2021/2022
800/961	White	B	Wildtype	FYA/FYB	Fy(a+b+)	Fya+Fyb+	2021/2022
798/978	Mixed	B	Wildtype	FYA/FYB^{ES}	Fy(a+b-)	Fya+	2021/2022
412/985	Black	O	Wildtype	FYB^{ES}/FYB^{ES}	Fy(a-b-)	No antigen	2021/2022
949/278	White	O	Wildtype	FYA/FYA	Fy(a+b-)	Fya+	2021/2022
796/301	Mixed	A	Wildtype	FYA/FYB	Fy(a+b+)	Fya+Fyb+	2021/2022
297/302	Black	A	Wildtype	FYB^{ES}/FYB^{ES}	Fy(a-b-)	No antigen	2021/2022
947/303	White	O	Wildtype	FYB/FYB*	Fy(a-b+)	Fyb+	2021/2022
416/557	White	A	Wildtype	FYA/FYB	Fy(a+b+)	Fya+Fyb+	2021/2022
415/558	White	O	Wildtype	FYB/FYB	Fy(a-b+)	Fyb+	2021/2022
559	White	A	Wildtype	FYA/FYB	Fy(a+b+)	Fya+b+	2022
**300/576**	**Black**	**O**	**Het**	**FYB^{ES}/FYB^{ES}**	**Fy(a-b-)**	**No antigen**	**2021/2022**
**038**	**Black**	**B**	**Het**	**FYB^{ES}/FYB^{ES}**	**Fy(a-b-)**	**No antigen**	**2022**
299/045	White	A	Wildtype	FYA/FYB	Fy(a+b+)	Fya+Fyb+	2021/2022
797/059	Asian (East)	O	Wildtype	FYA/FYA	Fy(a+b-)	Fya+	2021/2022
060	Black	O	Wildtype	FYB/FYB^{ES}	Fy(a-b+)	Fyb+	2022
799	Black	O	Hom	FYB^{ES}/FYB^{ES}	Fy(a-b-)	No antigen	2021
413	Asian (South)	AB	Wildtype	FYA/FYX2	$Fy(a+b^{weak})$	$Fya+Fyb^{weak}$	2021
414	Other	O	Wildtype	FYB/FYB	Fy(a-b+)	Fyb+	2021
298	Mixed	O	Wildtype	FYA/FYB	Fy(a+b+)	Fya+Fyb+	2021
301	White	A	Wildtype	FYB/FYB	Fy(a-b+)	Fyb+	2021
946	Black	O	Het	FYB^{ES}/FYB^{ES}	Fy(a-b-)	No antigen	2021
948	White	O	Wildtype	FYB/FYB	Fy(a-b+)	Fyb+	2021

1. A total of 20 donors were sampled in 2021 (round 1), and 13 of them were re-sampled again in 2022 (round 2). Three new donors (559, 038, and 060) were added in 2022 to make up for the 2021 donors that were not available in 2022.
2. Sample ID column represent the last 3 digits of the sample IDs for participants sampled in either 2021, 2022 or both years. For example, participants sampled/recalled in both 2021 and 2022 were give a unique sample ID for each year (round) which is separated by "/".
3. In the rare FYX2 allele, the presence of the 298G>A and 265C>T can decrease the expression of Fyb+ antigen resulting in a weak phenotype (Olsson et al. 1998), as is the case with sample 413, highlighted in blue above.
4. All ethnicities are self-reported; and *ES* = erythrocyte silent (i.e., lack of Fy antigen expression only on RBCs)
5. The expression of ABCA7 was downregulated in the Duffy-negative RBC samples (Figure 3), thus all samples were genotyped for a 44bp deletion (rs142076058) that disrupt the expression of the protein, we found three (3) heterozygous (3003/576, 038, 946) and one (1) homozygous samples with the deletion, however, the deletions does not correlate with protein expression as the relative abundance of ABCA7 was also decreased in samples that are wildtype for the deletion, as shown in Figure 3A.

Table S3: Distribution of Duffy genotypes and phenotypes in our study population

Genotype	Phenotype	Asian	Black	Caucasian	Mixed/ Other	Number of Samples
FYA/FYA	Fy(a+b-)	1	0	1	0	2
FYB/FYB	Fy(a-b+)	1	0	4	1	6
FYA/FYB	Fy(a+b+)	0	0	4	2	6
FYA/FYB^{ES}	Fy(a+b-)	0	0	0	1	1
FYB/FYB^{ES}	Fy(a-b+)	0	1	0	0	1
FYB^{ES}/FYB^{ES}	Fy(a-b-)	0	6	0	0	6
FYA/FYX2	$Fy(a+b^{weak})$	1	0	0	0	1
	**Total**	3	7	9	4	23

Table S4. Differentially expressed proteins between Duffy-positive and -negative RBCs

Accession	Gene	Protein name	Peptides	*P* value	Relative abundance in Duffy-negative
Q16570	ACKR1	Atypical chemokine receptor 1	1	$p<0.0001$	down
Q8IZY2	ABCA7	ATP binding cassette subfamily A member 7	14	$P<0.0001$	down
P22234	PAICS	Isoform 2 of Multifunctional protein ADE2	1	$p<0.05$	down
P11279	LAMP1	Lysosome-associated membrane glycoprotein 1	2	$p<0.05$	down
O95747	OXSR1	Serine/threonine-protein kinase OSR1	3	$p<0.05$	down
P11498	PC	Pyruvate carboxylase	17	$p<0.05$	down
P08575	PTPRC	Receptor-type tyrosine-protein phosphatase C	7	$p<0.05$	down
P62333	PSMC6	26S protease regulatory subunit 10B	1	$p<0.05$	down
P05026	ATP1B1	Sodium/potassium-transporting ATPase subunit beta-1	2	$p<0.05$	down
P10321	HLA-C	HLA class I histocompatibility antigen, Cw-17 alpha	1	$p<0.05$	down
P07355	ANXA2	Isoform 2 of Annexin A2	1	$p<0.05$	down
Q12913	PTPRJ	Receptor-type tyrosine-protein phosphatase eta	2	$p<0.05$	down
A6NFX1	MFSD2B	Major facilitator superfamily domain-containing protein 2B	2	$p<0.05$	up
Q02094	RHAG	Rh-associated glycoprotein	2	$p<0.05$	up
P78417	GSTO1	Glutathione S-transferase omega-1	1	$p<0.05$	up
O15439	ABCC4	Multidrug resistance-associated protein 4	41	$p<0.05$	up
P04156	PRNP	Major prion protein	4	$p<0.05$	up
P0C0L5	C4B	Complement C4-B (Chido blood group)	2	$p<0.05$	up
P02786	TFRC	Transferrin receptor protein 1	3	$p<0.005$	up

The table shows all (19) differentially expressed proteins between Duffy-positive and -negative RBCs as quantified by Plasma membrane profiling-based mass spectrometry. Proteins whose abundance is decreased in Duffy-negative relative to the Duffy-positive RBCs are highlighted in blue, while those with increased abundance are highlighted in light grey. A two-tailed t-test was used to determine the level of significance and estimate *P* values, which were corrected for multiple hypothesis testing using the Benjamini–Hochberg method.

Table S5: Effect of O blood group on *P. falciparum* invasion of Duffy RBCs.

Variable	Estimate	SE	*P value*	Summary
***P. falciparum* (3D7)**				
Fy phenotype[Fy(a+b+)]	1.52	4.67	0.75	Not significant
Fy phenotype[Fy(a+b-)]	-1.59	4.46	0.73	Not significant
Fy phenotype[Fy(a-b-)]	-6.55	4.09	0.14	Not significant
ABO[B]	-0.927	4.35	0.84	Not significant
ABO[O]	-0.322	4.16	0.94	Not significant
Adjusted R^2*=0.02*				
***P. falciparum* (Dd2)**				
Fy phenotype[Fy(a+b+)]	-4.03	4.25	0.36	Not significant
Fy phenotype[Fy(a+b-)]	-5.11	4.06	0.24	Not significant
**Fy phenotype[Fy(a-b-)]**	**-10.6**	**3.72**	**0.02**	**Significant**
ABO[B]	-4.13	3.95	0.32	Not significant
ABO[O]	0.37	3.79	0.92	Not significant
Adjusted R^2*=0.33*				
P. knowlesi				
Fy phenotype[Fy(a+b+)]	-2.79	6	0.65	Not significant
Fy phenotype[Fy(a+b-)]	-7.06	5.74	0.25	Not significant
**Fy phenotype[Fy(a-b-)]**	**-28.3**	**5.26**	**<0.001**	**Significant**
ABO[B]	-0.823	5.59	0.89	Not significant
ABO[O]	1.46	5.35	0.79	Not significant
Adjusted R^2*=0.70*				

To rule out any potential confounding effect of the O blood group on the invasion data, we performed a multiple linear regression (MLR) analysis that included the ABO genotypes of the study participants. The analysis showed that the ABO blood groups had no statistically significant impact on the invasion data. However, the Fy(a-b-) phenotype had a minor effect on 3D7 invasion, albeit not statistically significant (*P=0.14*), but had a significant effect on Dd2 invasion (*P=0.02*). As expected, the largest effect of the Duffy-negative phenotype on invasion was noted on the *P. knowlesi* parasites (*P<0.001*). The statistically significant differences are highlighted in bold, and the following blood groups were used as reference: ABO blood group = A, and Duffy phenotype = Fy(a-b+). Adjusted R^2 = coefficient of determination, and SE= standard error of the estimate.

Table S6: FYB^{ES} and sickle cell frequencies in African populations

Population	Location	HbS frequency	FYB^{ES} frequency	Longitude	Latitude	Subgroup
Mossi	Burkina Faso	0.034181	1	-1.56159	12.23833	North
Bantu	Cameroon	0.082452	0.99498	12.35472	7.369722	North
Semi-Bantu	Cameroon	0.063985	0.994737	12.35472	7.369722	North
Fula	Gambia	0.068069	0.998244	-15.3101	13.44318	North
Jola	Gambia	0.032138	0.99927	-15.3101	13.44318	North
Mandinka	Gambia	0.059532	0.998706	-15.3101	13.44318	North
Wollof	Gambia	0.044461	0.999302	-15.3101	13.44318	North
Akans (Ashanti Eastern)	Ghana	0.037736	0.99697	-1.02319	7.946527	North
Kasem	Ghana	0.022267	1	-1.02319	7.946527	North
Nankam	Ghana	0.029586	1	-1.02319	7.946527	North
Northerner	Ghana	0	1	-1.02319	7.946527	North
Bambara	Mali	0.02451	1	-3.99617	17.57069	North
Malinke	Mali	0.034091	1	-3.99617	17.57069	North
Peulh	Mali	0.04	1	-3.99617	17.57069	North
Sarakole	Mali	0.037037	1	-3.99617	17.57069	North
Yoruba	Nigeria	0.069565	1	8.675277	9.081999	North
Baya-Mandja (Spedini et al. 1981)	Central African Republic	0.0435	0.8528	21.36	4.77	SouthCentral
Mbugu (Spedini et al. 1981)	Central African Republic	0.0741	0.8841	21.36	4.77	SouthCentral
Sango (Spedini et al. 1981)	Central African Republic	0.1227	0.8047	21.36	4.77	SouthCentral
Yakpa (Spedini et al. 1981)	Central African Republic	0.0694	0.8669	21.36	4.77	SouthCentral
Nyaturu (Godber et al. 1976)	Tanzania	0.037	0.9908	35.7	-4.9	SouthEast
Sandawe (Godber et al. 1976)	Tanzania	0.0138	0.9906			SouthEast
Chonyi	Kenya	0.083167	0.998972	37.90619	-0.02356	SouthEast
Giriama	Kenya	0.035341	0.996289	37.90619	-0.02356	SouthEast
Kauma	Kenya	0.076531	1	37.90619	-0.02356	SouthEast
Malawi	Malawi	0.018003	0.994754	34.30153	-13.2543	SouthEast
Mzigua	Tanzania	0.053571	0.997449	34.88882	-6.36903	SouthEast
Wabondei	Tanzania	0.043956	1	34.88882	-6.36903	SouthEast
Wasambaa	Tanzania	0.038012	0.997076	34.88882	-6.36903	SouthEast
Kgalagadi (Jenkins et al. 1987)	Botswana (Kweng District)	0	0.835	24.70245	-23.87	Southwest
Gciriku (Nurse and Jenkins 1977)	Southwest Africa	0.052	0.923	19.8	-17.91	Southwest

Kwangali (Nurse and Jenkins 1977)	Southwest Africa	0.034	0.905	19.8	-17.91	Southwest
Mbukushu (Nurse and Jenkins 1977)	Southwest Africa	0	0.96	19.8	-17.91	Southwest
Sambyu (Nurse and Jenkins 1977)	Southwest Africa	0.082	0.93	19.8	-17.91	Southwest
Kwengo (Nurse and Jenkins 1977)	Southwest Africa	0	0.885	19.8	-17.91	Southwest

Sources are anthropological studies, as indicated in the references, and MalariaGEN SNP data (rs334 in beta globin and rs2814778 in DARC (Malaria Genomic Epidemiology Network et al. 2019); for simplicity, we assume that all instances of rs2814778 "G" represent FYB^{ES}, which is a reasonable assumption for African populations). For study (Nurse and Jenkins 1977), the sickle cell allele frequency calculation assumes the number of sickle cell individuals reported is the number of heterozygotes (we believe there to have been a typo in table 9 of study (Nurse and Jenkins 1977) wherein the reported sickle cell allele frequencies of the Sambyu and Gciriku populations are incorrect and we have calculated our own allele frequency values based on the data in the rest of table 9 of that paper).

Table S7: Parameters of the evolutionary-epidemiological model

Parameter	Definition	Value used	Notes
μ	Death rate of hosts from causes other than malaria	1/25	mean lifespan of a host = 25 years (intended to be plausible for the period of history over which the evolution of Duffy negativity is likely to have occurred)
σ	Recovery rate from malaria infection	2	mean duration of infection = 6 months (mean durations of infection assumed for *P. falciparum* malaria in epidemiological models range from 20 to 200 days (Mandal et al. 2011); 6 months is in keeping with that assumed by (Filipe et al. 2007), based on 20th century malaria therapy studies.
β	Transmission parameter, related to the basic reproduction number (R_0) in this model as follows: $R_0 = \frac{\beta}{\sigma+\mu+\alpha_1+\alpha_2}$	Values between 3 and 11 were tested.	β values were chosen such that R_0 takes values between 1.7 and 5.
α_1	Severe malaria mortality rate (applies to virulent infections, before immunity to virulence is gained)	0.01	For the values of θ, σ, and *r* in this table, if R_0 =1.7, these combined mortality rates mean malaria deaths each year are equal to 4.2% of the births. If R_0=3, these combined mortality rates mean malaria deaths each year are equal to 4.93% of the births. If R_0=5, 5.19% of the births; if R_0=10, 5.25% of the births each year. This non-linear relationship between R_0 and the cost of malaria to the population occurs because adaptive immunity to the worst virulent effects of malaria is possible within the model, and always gained after the same average number of infections.
α_2	Baseline malaria mortality rate (applies to all malaria infections, regardless of immunity to virulence)	0.00001	
ψ	Reproductive cost to the host of severe infection with malaria	1	Severe infection is associated with a 100% probability of reproductive failure (i.e. severe infection whilst pregnant would lead to loss of pregnancy or stillbirth). This figure looks high, but as discussed in (Penman and Gandon 2020), this figure is supposed to capture the reproductive costs of experiencing *P. falciparum* malaria whilst pregnant without any prior adaptive immunity, which is known to be very dangerous. If transmission is high, individuals gain virulence immunity long before reaching reproductive maturity, and do not experience this cost.
g	Rate at which hosts become reproductively mature	1/15	Mean time to reach reproductive maturity = 15 years.
K	Carrying capacity of population	10000	

r	Fecundity parameter, related to the birth rate as defined in equation 10.	0.2	
**θ**	Probability of a host gaining immunity to the virulent effects of malaria upon recovery from infection.	1/15	This assumes that the average number of infections before gaining immunity to virulence is 15. It is well established that individuals do gain immunity to severe malaria after repeated infection, but the number of infections that is necessary to achieve this is unknown. Figure S9 presents a sensitivity analysis of the model in which we show that the same broad pattern emerges with different values of θ.
p_i	Proportion of infections blocked for genotype *i*	Always 0 for the wild type genotype, values for FYB^{ES} homozygotes and heterozygotes based on experimental results from this paper.	
q_i	Protection against severe (virulent) malaria mortality enjoyed by genotype *i*.	Always 0 for the wild type genotype, no protection associated with FYB^{ES}, but sickle cell heterozygotes are 91% protected against death from severe malaria, based on (Taylor et al. 2012).	
w_i	Protection against baseline malaria mortality enjoyed by genotype *i*.	Always 0 for the wild type genotype, no protection associated with FYB^{ES}, but sickle cell heterozygotes are 31% protected against baseline malaria mortality, based on (Taylor et al. 2012).	

References

Filipe JAN, Riley EM, Drakeley CJ, Sutherland CJ, Ghani AC. 2007. Determination of the processes driving the acquisition of immunity to malaria using a mathematical transmission model.De Boer RJ, editor. *PLoS Comput. Biol.* 3:e255.

Godber M, Kopeć AC, Mourant AE, Teesdale P, Tills D, Weiner JS, El-Niel H, Wood CH, Barley S. 1976. The blood groups, serum groups, red-cell isoenzymes and haemoglobins of the Sandawe and Nyaturu of Tanzania. *Ann. Hum. Biol.* 3:463–473.

Jenkins T, Speirs J, Dunn DS, Nurse GT. 1987. Serogenetic and haematological studies on the Kgalagadi of Botswana. *Ann. Hum. Biol.* 14:143–153.

Malaria Genomic Epidemiology Network, Band G, Le QS, Clarke GM, Kivinen K, Hubbart C, Jeffreys AE, Rowlands K, Leffler EM, Jallow M, et al. 2019. Insights into malaria susceptibility using genome-wide data on 17,000 individuals from Africa, Asia and Oceania. *Nat. Commun.* 10:5732.

Mandal S, Sarkar R, Sinha S. 2011. Mathematical models of malaria - A review. *Malar. J.* 10:202.

Nurse GT, Jenkins T. 1977. Serogenetic studies on the Kavango peoples of South West Africa. *Ann. Hum. Biol.* 4:465–478.

Olsson ML, Smythe JS, Hansson C, Poole J, Mallinson G, Jones J, Avent ND, Daniels G. 1998. The Fy^x phenotype is associated with a missense mutation in the Fy^b allele predicting Arg89Cys in the Duffy glycoprotein. *Br. J. Haematol.* 103:1184–1191.

Penman BS, Gandon S. 2020. Adaptive immunity selects against malaria infection blocking mutations. *PLoS Comput. Biol.* 16:e1008181.

Spedini G, Capucci E, Rickards O, Fuciarelli M, Giaccaia L, Aebischer ML, Mannella E, Loreti O. 1981. Some genetic erythrocyte polymorphisms in the Mbugu and other populations of the Central African Republic with an analysis of genetic distances. *Anthropol. Anz.* 39:10–19.

Taylor SM, Parobek CM, Fairhurst RM. 2012. Haemoglobinopathies and the clinical epidemiology of malaria: A systematic review and meta-analysis. *Lancet Infect. Dis.* 12:457–468.

Theron M, Cross N, Cawkill P, Bustamante LY, Rayner JC. 2018. An in vitro erythrocyte preference assay reveals that *Plasmodium falciparum* parasites prefer type O over type A erythrocytes. *Sci. Rep.* 8:8133.
